# Niclosamide, an antihelmintic drug, enhances efficacy of PD-1/PD-L1 immune checkpoint blockade in non-small cell lung cancer

**DOI:** 10.1186/s40425-019-0733-7

**Published:** 2019-09-11

**Authors:** Fan Luo, Min Luo, Qi-Xiang Rong, Hong Zhang, Zhen Chen, Fang Wang, Hong-Yun Zhao, Li-Wu Fu

**Affiliations:** State Key Laboratory of Oncology in South China, Collaborative Innovation Center for Cancer Medicine, Guangdong Esophageal Cancer Institute, Sun Yat-sen University Cancer Center, Guangzhou, 510060 People’s Republic of China

**Keywords:** Niclosamide, PD-L1, STAT3, Immunotherapy, NSCLC

## Abstract

**Background:**

PD-1/PD-L1 blockade has received approval for clinical application due to its encouraging benefit with improving prognosis in selected populations. Unfortunately, the response to immunotherapy for many patients remains unsatisfactory. It remains a great challenge to generate potential combinations that will outperform single agents alone with regard to anti-tumor activity.

**Methods:**

Using NSCLC cell lines and mouse models, we explored the effects of combined niclosamide and PD-L1 blockade on tumor growth and T cell function. Furthermore, we investigated the relationship between PD-L1 and p-STAT3 expression in tumor samples from patients with NSCLC using IHC, as well as their relationship to patient survival.

**Results:**

In vitro, niclosamide, an antihelmintic drug, enhanced the cancer cell lysis mediated by T cells in the presence of PD-L1 blockade. Accordingly, mice treated with niclosamide and PD-L1 antibody showed significant delay in tumor growth and increased survival which were associated with the increase of tumor infiltrating T cells and granzyme B release. Importantly, we found niclosamide could decrease the expression of PD-L1 in both a concentration- and time-dependent manner in NSCLC cells, which was linked to the blockage of p-STAT3 binding to the promoter of PD-L1.

**Conclusions:**

An enhancement of PD-L1 antibody by niclosamide was observed in inhibition of NSCLC growth in vitro and in vivo, which was involved in blockage of p-STAT3 binding to promoter of PD-L1 and finally downregulation of PD-L1 expression. These encourage the combination therapy of niclosamide and PD-1/PD-L1 blockade to be further studied in clinic.

**Supplementary information:**

**Supplementary information** accompanies this paper at 10.1186/s40425-019-0733-7.

## Introduction

Non-small cell lung cancer (NSCLC) is the leading cause of cancer mortality and has poor prognosis [1]. In recent years, advances in the treatment of NSCLC have been substantial and promising with the effective application of immunotherapies, including anti-programmed cell death 1 ligand (PD-L1) and anti-programmed cell death 1 (PD-1) antibodies (nivolumab, atezolizumab and pembrolizumab), in selected populations of advanced NSCLC with high tumor mutation burden (TMB) or elevated pretreatment PD-L1 expression [2]. Immune checkpoint blockades, particularly targets of co-inhibitory pathways in T cells, can enhance the anti-tumor immune response [3]. Basing on the results of some phase III clinical trials, the Food and Drug Administration (FDA) has approved PD-1/PD-L1 inhibitors to be used in first- or second-line treatment of patients with advanced NSCLC [4]. Despite encouraging results with prolonged survival in some patients, only approximately 20% of NSCLC patients can effectively respond to immune checkpoint inhibitor as monotherapy due to the complexity of tumor immune microenvironment [5] . It is of serious need to explore potential combination therapies to enhance the efficacy of immune checkpoint inhibitors.

Some clinical trials about combining anti-PD-1/PD-L1 with anti-CTLA-4 have obtained positive results in various cancers, including melanoma [6] and NSCLC [7]. The CheckMate 067 has demonstrated that the objective response rate is higher than single agents (57.6% of nivolumab plus ipimumab vs 43.7% of nivolumab alone or 19.0% of ipimumab alone), but the incidence of treatment-related adverse events of grade 3 or 4 in the combined group is also higher than monotherapies (55.0% of nivolumab plus ipimumab vs 16.3% of nivolumab alone or 27.3% of ipimumab alone) in melonama in phase III clinical trial [6]. Although the promising therapeutic effects of the aforementioned combination therapy have been proved in some phase I or II clinical studies, the phase III MYSTIC trial has not observed positive results in the treatment of NSCLC patients [8]. Elevated indoleamine 2, 3-dioxygenase(IDO) is positively correlated with increased tumor grade, peripheral immune tolerance and poor prognosis in various cancers [9]. Despite previous encouraging response of the IDO inhibitors in combination with anti-PD-1 in many different solid tumors was observed in preclinical investigations and early-phase clinical studies, a phase III clinical trial demonstrated negative results with regard to such a combination treating metastatic melanoma [10]. So to find the novel combination regimen with PD-1/PD-L1 blockage is a promising approach to improve the therapeutic reaction of advanced NSCLC.

PD-L1 expression is related to poor prognosis for patient survival in several tumor types [11]. High levels of PD-L1 have been also reported to be related to resistance to anti-tumor therapies and to be involved in the process of immune escape [12]. Inhibition of the PD-1/PD-L1 pathway enhances the amplitude of anti-cancer immunity in NSCLC [13]. More importantly, a correlation has been observed between tumor expression of PD-L1 and the likelihood of effectiveness of PD-1/PD-L1 blockade in NSCLC [14] and melanoma [15], suggesting that PD-L1 expression in tumor cells may predict or be related to the response to anti-PD-1/PD-L1 therapies. Activation of the JAK/STAT3 pathway is correlated with upregulation of PD-L1 expression in diverse cancer types including lymphoma [16] and head and neck squamous cell carcinoma (HNSCC) [17]. Thus, therapeutic approaches targeting the JAK/STAT3 pathway may benefit cancer patients, not only through promoting tumor inhibition but also by attenuating PD-L1 expression to enhance anti-tumor immunity. Previous studies have shown that combined use of ruxolitinib, a JAK inhibitor, with a PD-L1 antibody (Ab) can overcome the resistance to PD-L1 Ab in an in vivo pancreatic xenograft model [18].

Niclosamide, approved by FDA for its antihelmintic efficacy, is a cell-permeable salicylanilide and can exert uncoupling effects to cause conformational transitions in mitochondrial catalytic proteins, further to kill the tapeworm [19, 20]. Beyond the approval treatment for parasitic disease, niclosamide has presented preclinical activity in various diseases, including cancers and infection and metabolic diseases etc. [21]. Recently, a new study has revealed that niclosamide can induce metabolic stress in p53 mutant colon cancer and further lead to cell death, showing that niclosamide may be used as a broad-spectrum drug to treat multiple cancers with p53 dysfunction [22]. Therefore, a phase II clinical trial in colorectal cancer has identified the potent anti-cancer activity of niclosamide with an acceptable safety profile [23], additionally, five clinical studies (NCT03123978; NCT02687009; NCT02807805; NCT02519582; NCT03521232) are ongoing to evaluate the efficacy of niclosamide against cancer.

In the current study, we identify that niclosamide in combination with PD-1/PD-L1 antibody can act a synergistic anti-tumor effect in vitro and in vivo for treatment of NSCLC through decreasing PD-L1 expression and further promoting cytotoxic T cell activity and enhancing antitumor immune responses. Furthermore, we demonstrate that PD-L1 downregulation induced by niclosamide is related to the inhibition of STAT3 phosphorylation and its binding to PD-L1 promoter. Our findings establish an alternative strategy targeting PD-L1 to improve immunotherapeutic efficacy by repurposing existing antitapeworm drug.

## Methods

### Chemicals and reagents

The following compounds and reagents were used: niclosamide was purchased from Selleck Chemicals, USA, and prepared in dimethyl formamide (DMF) at a concentration of 5 mmol/L and maintained at − 20 °C. Niclosamide was diluted in DMF for working solutions and used at concentrations ranging from 0.25 μmol/L to 2 μmol/L for the treatment of cells. The following antibodies were purchased from Cell Signaling Technology, USA: phospho-STAT3 (p-STAT3), STAT3. Human B7H1/PD-L1 polyclonal antibody was purchased from Santa Cruz Biotechnology, USA, and GAPDH antibody was from Abcam, UK. Polyclonal goat anti-mouse antibody and goat anti-rabbit antibody (R&D systems, USA) were used for Western blotting.

### Cell lines and cell culture

NSCLC cell lines (A549, H1299, H460), Lewis lung cancer (LLC) cell line, and 293 T cell line were obtained from the American Type Culture Collection (ATCC, USA) and validated by short-tandem-repeat (STR) analysis (except for LLC). Cells were cultured in either RPMI-1640 (for NSCLC cell lines) or DMEM (for LLC cells and 293 T cells) containing 10% fetal bovine serum and maintained at 37 °C in a humidified 5% CO_2_ incubator. Peripheral blood mononuclear cells (PBMCs) were cultured in T cell medium (RPMI-1640 supplemented with 10% human serum, 5% L-glutamine-penicillin-streptomycin solution (Sigma-Aldrich, USA), and IL-2 (100 IU/mL).

### Cell cytotoxic assay

Cytotoxicity studies were performed using the thiazolyl blue tetrazolium bromide (MTT) assay. MTT assay was performed to examine sensitivity of the cells to niclosamide as described previously [24]. Experiments were conducted at least three times. The concentration of niclosamide suppressing cell proliferation by 30% (IC30), calculated from survival curves using the Bliss method, was selected for further experiments.

### Western blot analysis

Cells were treated with the indicated concentrations as shown in the figures and washed twice with cold PBS. Whole cell extracts were collected in RIPA lysis buffer (Santa Cruz Biotechnology, Germany), and protein concentration of the lysates was measured using a BCA Protein Assay Kit (Pierce Biotechnology, USA). The protein samples were electrophoresed through a 10% SDS-PAGE gel and transferred to a polyvinylidene difluoride (PVDF) membrane (Millipore, USA). After blocking, membranes were probed with primary antibodies (1:1000) followed by washing and incubation with a secondary antibody (1:5000) conjugated to horseradish peroxidase (Amersham GE Healthcare, USA). Protein bands were visualized by applying a chemiluminescent reagent (Pierce ECL kit, Thermo Fisher Scientific, USA).

### RNA extraction and quantitative real-time PCR

Total cellular RNA was isolated using Trizol (Life Technologies, USA) according to the manufacturer’s protocol. For first-strand cDNA synthesis, 5 μg of total RNA was reverse-transcribed using the GoScript™ Reverse Transcription System kit (Promega, USA) followed by quantitative polymerase chain reaction (qPCR) with GoTaq qPCR Master Mix (Promega, USA), according to the manufacturer’s instructions. Real-time PCR analyses were conducted using the Biorad CFX96 system with SYBR green (Bio-Rad, USA) and the appropriate primers to estimate the mRNA expression levels of *STAT3* and *PD-L1*. Data were normalized to *GAPDH* levels. Experiments were performed in triplicates. The primes are as follows: Stat3 forward: CTTGACACACGGTACCTGGA; reverse: CTTGCAGGAAGCGGCTATAC; PDL1 forward: TATGGTGGTGCCGACTACAA; reverse: TGCTTGTCCAGATGACTTCG; β-actin forward: TCCTGTGGCATCCACGAAACT; reverse: GAAGCATTTGCGGTGGACGAT.

### Transfection of shRNA and plasmid DNA

STAT3 shRNAs and a shRNA scramble control (Additional file 1: Table S1) (Open Biosystems GE Healthcare Dharmacon Inc., USA) were transiently transfected along with a pSIH-H1-puro Lentivector Packaging Kit (System Biosciences, USA). Transfections were carried out in 293 T cells grown to ∼80% confluency in 10 cm dishes using Lipofectamine 2000 transfection reagent (Life Technologies, USA) and following the manufacturer’s instructions. H460 and H1299 cells were infected and incubated with the viral particles overnight at 37 °C. At 48 h after transfection, cells were placed under puromycin selection by supplementing the growth medium with puromycin (3 μg/ml for H460, and 4 μg/ml for H1299). Stable repression of gene expression was verified by Western blotting and RT-PCR.

### Dual-luciferase reporter assay

An 868-bp PD-L1 promoter fragment (UCSC: http://genome.ucsc.edu/, the gene ID: 29126) (nucleotides − 762 to + 106 base pair (bp) relative to the translation initiation site) was PCR-amplified from H460 cell line genomic DNA and inserted into the promoter-less plasmid pGL3-Basic (Promega, USA), designated as p868. A series of 5′-deletions were produced by PCR using p868 as a template with the distinct 5′ primers a common 3′ primer (Additional file 1: Table S2). The products were cloned into pGL3-Basic to generate p693, p516, and p360. The promoter sequences were then interrogated for transcription factor binding sites and transcription factor modules with the use of PROMO (http://alggen.lsi.upc.es/) and the JASPAR database (http://jaspar.genereg.net). The STAT3 cDNA was PCR amplified with the relevant primers (Additional file 1: Table S2) and cloned into the plasmid PCDNA3.1 (Promega, USA). The 293 T cell lines were grown to approximately 80% confluence, and 4 × 10^5^ cells each were co-transfected with 3.8 μg/well of pGL3 luciferase construct (empty vector or pGL3-PD-L1promoter) and 0.2 μg/well pRL-TK (Promega, USA). The relative luciferase activity was examined by Dual Luciferase Assay Kit (Promega, Madison, WI, USA) in accordance with the manufacturer’s protocols.

### Colony formation assay

As effector cells, human PBMCs were purified from the blood of healthy volunteers using Ficoll gradient centrifugation (Solarbio, Beijing). The purity of the isolated cells was > 95%, as determined in flow cytometry (FCM). Briefly, 24-well plates were coated overnight with 5 μg/ml anti-CD3 (BD Bioscience, USA), then washed twice with PBS. PBMCs were plated in complete TCCM medium (IMDM with human AB serum (5%), penicillin–streptomycin, HEPES, 2-mercaptoethanol, and gentamicin). As target cells, cancer cells were pre-treated with niclosamide (2 μmol/L) for 24 h; control cells were without niclosamide pre-treatment. Then, cells were treated with PD-L1 Ab or not and co-cultured with activated PBMCs at several target-to-effector ratios (1:0, 1:1, 1:4, 1:16) (all samples in triplicate). After 4 days of co-incubation, 24-well plates wells were washed with PBS twice to remove PBMCs and then the survived tumor cells were fixed and stained with Giemsa staining solution. The dried plates were scanned and quantified the intensity.

### Flow cytometry analysis

6-well plates were coated overnight with 5 μg/ml anti-CD3 (Biolegend, USA), then washed twice with PBS. PBMCs were plated at a density of 1 × 10^6^/well in 6-well plates and then co-cultured with tumor cells pre-treated with niclosamide at 4:1 ratio for 24 h. Anti-human PD-L1 antibody, atezolizumab (Selleck Chemicals, USA) (50 μg/ml), was added to the appropriate wells. After co-culturing, the PBMCs were isolated and stained with anti-CD3 and anti-CD8 antibodies to estimate the CD8+ cell proportions. For TNF-α and granzyme B analysis, PBMCs were harvested and then treated with brefeldin A (Biolegend, USA) at 37 °C for an additional 3 h to prevent extracellular secretion. Subsequently, PBMCs were fixed and permeabilized with the Intracellular Fixation and Permeabilization Buffer Set Kit (eBioscience, USA) following the manufacturer’s instructions. Then percentages of TNF or Granzyme B positive cells in CD3+ T cells or CD8+ T cells were labeled via intracellular staining and detected by flow cytometry. Antibodies for flow cytometry analysis were purchased from eBiosciences, USA. Matched isotype controls were used for each antibody to determine gates. FlowJo (Treestar, USA) software was used for the analysis of flow cytometry data. Standardized fluorescence intensities were calculated by dividing the median fluorescence intensities of specific antibodies by the median fluorescence intensities of isotype controls. The results are expressed as mean ± SD of three independent experiments.

### In vivo mouse studies

C57BL/6 mice were obtained from Guangdong Medical Laboratory Animal Center, China, and kept in a specific pathogen-free (SPF) barrier facility at the Animal Center of Sun Yat-sen University Cancer Center. The female mice with 8–12 weeks old were used for all animal experiments. Experiments were approved by the institutional committee of Sun Yat-sen University Cancer Center, and conducted in accordance with protocols approved by the Guangdong Provincial Animal Care and Use Committee.

LLC cells (2 × 10^5^ cells in 200 μL growth medium) were subcutaneously injected into the right flank of immunocompetent C57BL/6 mice. Tumor growth was measured with calipers every 3 days and the tumor volumes were calculated by applying the following formula: 1/2(length x width^2^). When tumors reached approximately 100 mm^3^, mice were randomized into control or experimental groups. A terminal event was defined as tumors reaching a size of 2000 mm^3^, at which point animals were euthanized [25, 26].

Mice were treated with niclosamide or rat anti-PD-L1 antibody (αPD-L1, clone 10F.9G2; BioLegend, USA) alone, the combination of niclosamide and αPD-L1, or saline and IgG2bκ (clone RTK4530; BioLegend, USA) by intraperitoneal injection (each group, *n* = 6–7). Niclosamide (20 mg/kg) or saline was administered intraperitoneally from day 13, every 5 days, after tumor implantation. Anti-PD-L1 antibody therapy (10 mg/kg) was administered intraperitoneally weekly on days 16, 23, 30, 37, and 44. Survival analysis was performed using Kaplan-Meier analysis and log-rank test.

### Patients and tissue specimens

Tissue specimens were obtained from 28 patients with advanced NSCLC who received immunotherapy during the course of anti-cancer therapy at Sun Yat-sen University Cancer Center (Guangzhou, China). Clinical data was collected from pathology reports and unprocessed medical files. The study was conducted with the permission of the Ethics Committee of the Sun Yat-sen University Cancer Institutional Board, and all patients involved provided informed, written consent.

### Histology and immunohistochemistry (IHC)

For IHC staining of the xenografts, tumor tissues were fixed, embedded, and sectioned (3 μm thick). Immunohistochemistry staining for human and mouse tissues was performed in accordance with standard procedures [27]. The following antibodies were used: primary antibody CD3+ (dilution 1:200) or Granzyme B (dilution 1:400) (Cell Signaling Technology, USA) for mouse tissues, and anti-PD-L1 (dilution 1:1000) (E1L3N, Cell Signaling Technology) or anti-p-STAT3 (dilution 1:500) (D3A7, Cell Signaling Technology) for human tissues.

### STAT3-niclosamide docking

Since no human STAT3 crystal structure has been elucidated at present, mouse STAT3 (PDB ID: 4e68) was selected as receptor for docking as the identity of key residues and the high sequence identity (76.5%) sharing between human and mouse STAT3. Residues Lys591, Arg609, Ser636, Glu638 and residues Val637, Ile653 constitute the phosphotyrosine binding site and hydrophobic binding site of STAT3, respectively. Thus, we defined residues 591, 609, 636–638, 653 as putative binding sites to investigate the binding of niclosamide to STAT3. The docking simulations were carried out using Surflex module of SYBYL software (Tripos, Inc), which combines Hammerhead’s empirical scoring function with a molecular similarity method (morphological similarity) to generate putative poses of ligand [28]. The crystal structure of STAT3 was retrieved from RCSB Brookhaven Protein Database (http://www.pdb.org/pdb/home/home.do) (PDB ID: 4e68). For molecular docking purpose, the substrate dsDNA M67 and crystal water molecules were removed, and all hydrogen atoms were subsequently added to the unoccupied valence of heavy atoms at the neutral state (pH 7). The small molecule niclosamide downloaded from pubchem database (https://pubchem.ncbi.nlm.nih.gov/) was employed to perform the docking process. Two parameters bloat and threshold, which determine how far a potential ligand should extend outside of the concavity and how deep into the protein the atomic probes, were used to define the protomol. For niclosamide, the protomol was generated using the residue approach, and the bloat and threshold were set to 0.4 and 1, respectively.

### Statistical analysis

Statistical analysis was carried out using IBM SPSS Statistics 19 software or GraphPad Prism using Student’s t-test or one-way ANOVA or Dunnett’s test. All experiments were repeated in triplicate. Data are expressed as mean ± standard deviation (SD). Statistical significance was defined as *P* < 0.05.

## Results

### Niclosamide potentiates anti-PD-L1 efficacy in vitro

**Fig. 1:**
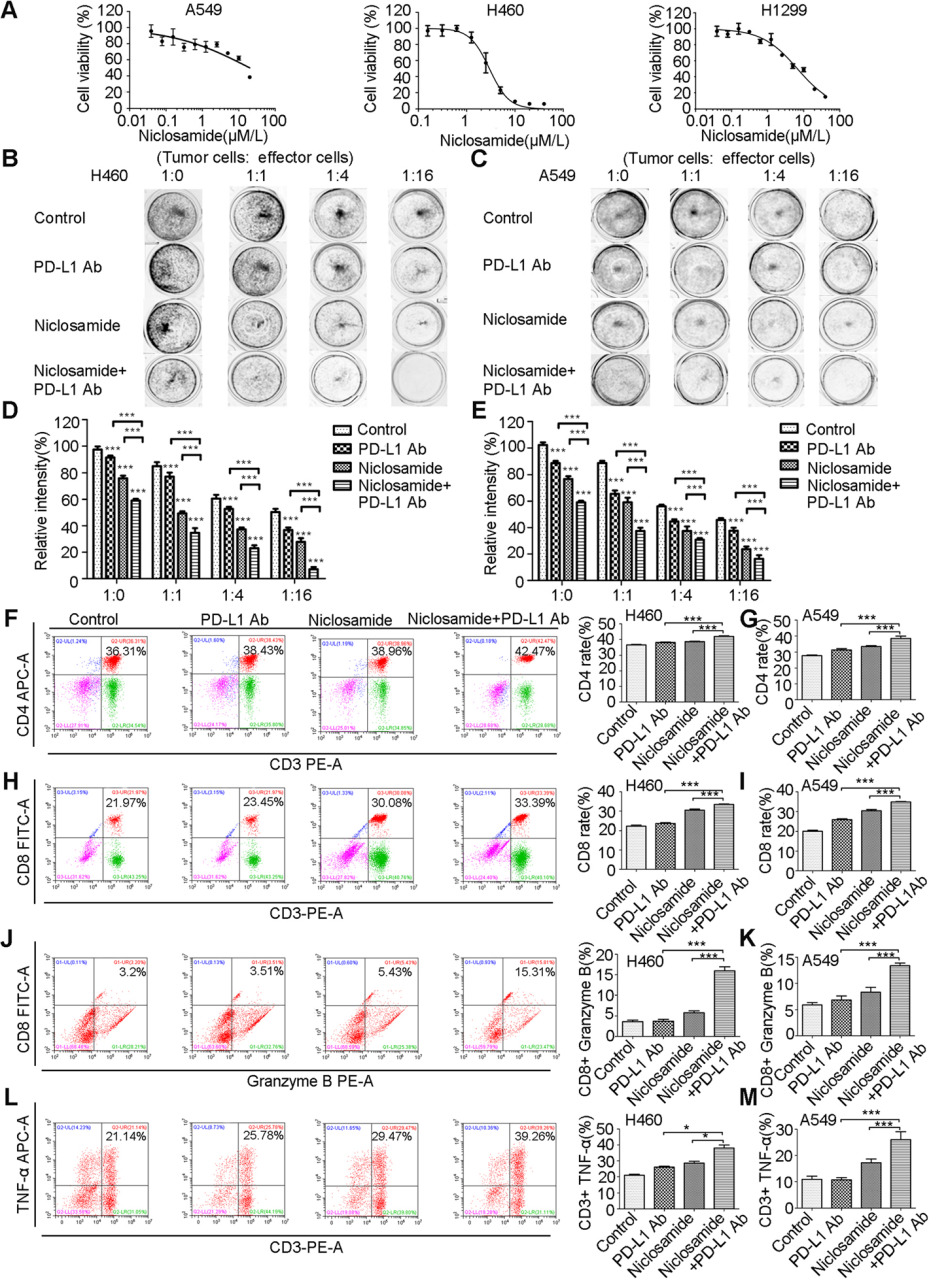
Niclosamide potentiates anti-PD-L1 efficacy in vitro. **a** The Cytotoxicity of niclosamide on different human cancer cells. Above assay were determined by MTT as described in materials and methods. Each point represents the mean ± standard deviations (SDs) of three independent experiments performed. **b**-**e** T cell cytotoxicity test by colony formation assay. The survival of niclosamide pretreated H460 and A549 cells, un-pre-treated cells, treated with PD-L1 Ab or without, and co-cultured with PBMCs (targeted cells: effector cells = 1:0, 1:1, 1:4, 1:16) in 24-well plates for 4 days was estimated. Colonies were visualized by Giemsa staining. Survival relative to control is shown. **f**-**i** The effect of different treatments on CD4+ and CD8+ populations among CD3+ T cells. Results are presented as means ± S.D. of a representative experiment performed in triplicate. **j**-**k** Intracellular cytokine staining of granzyme B in T cell-mediated tumor cell killing assay in niclosamide-pretreated H460 and A549 cells and un-pre-treated cells, treated with PD-L1 Ab or without. **l**-**m** Intracellular cytokine staining of TNF-α in T cell-mediated tumor cell killing assay in niclosamide-pretreated and un-pre-treated H460 and A549 cells, treated with PD-L1 Ab or without. Results are presented as means ± S.D. of a representative experiment performed in triplicate. * *P* < 0.05, ** *P* < 0.01, *** *P* < 0.001

First, in order to exclude any underlying bias caused by variation in growth suppression induced by the niclosamide, we performed growth inhibition curves for different cell lines and established an inhibitory concentration of 30% (IC30) (Fig. 1a). Then, to investigate whether niclosamide combined with PD-1/PD-L1 blockade can exert a synergistic immunotherapeutic effect, we tested the efficacy of the combined use of niclosamide and anti-PD-L1 blocking antibodies in vitro. Niclosamide combined with PD-L1 antibody (atezolizumab) showed significantly higher tumor growth inhibition compared to niclosamide alone or PD-L1 blockade alone (Fig. 1b-e). To further explore the cellular and molecular mechanisms involved in the therapeutic benefit of the combined treatment, we evaluated the proportion of CD3 + CD4+ and CD3 + CD8+ T cells using flow cytometry and observed the largest increase of CD3 + CD4+ and CD3 + CD8+ T cells in the combinational group (Fig. 1f-i). We also measured the production of TNF-α and granzyme B within the co-culturing system. Compared to the monotherapy or control groups, niclosamide augmented the release of TNF-α and granzyme B in response to anti-PD-L1 therapy (Fig. 1j-m). These findings show that niclosamide increases the number of functionally active CD8+ T cells and CD4+ T cells, finally potentiates anti-PD-L1 response.

### Niclosamide potentiates anti-PD-L1 efficacy in vivo

**Fig. 2:**
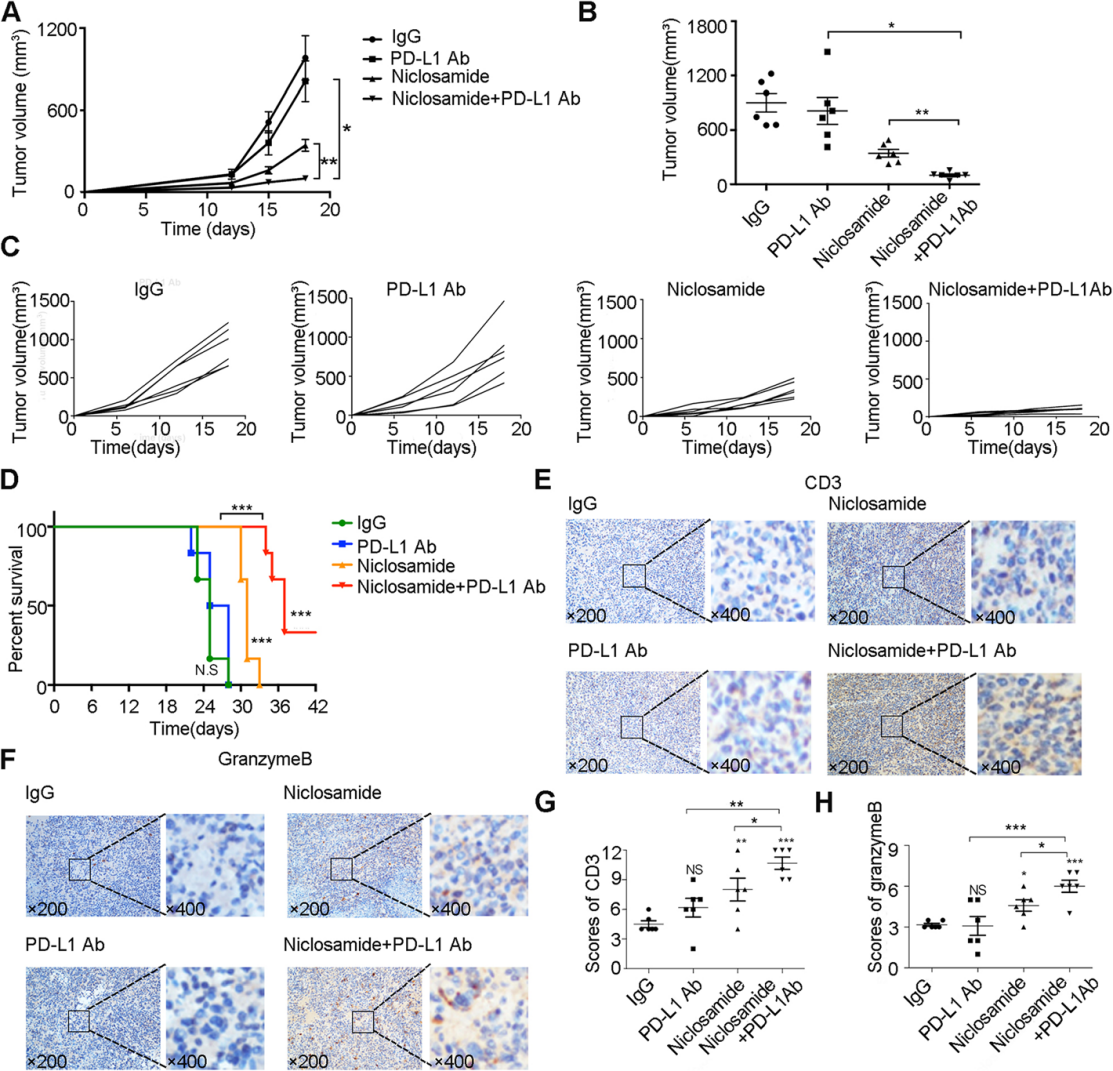
Niclosamide potentiates anti-PD-L1 efficacy in vivo. **a**-**c** Tumor volumes determined at the indicated days with different treatments in C57BL/6 mice (*n* = 6). Error bars represent SD of three independent experiments. **d** Survival analysis of C57BL/6 mice with different treatments (n = 6). **e**-**h** Immunohistochemistry staining of CD3 and Granzyme B in xenograft tumors (*n* = 6). * *P* < 0.05, ** *P* < 0.01, *** *P* < 0.001

In LLC cell tumor-bearing mice, mice receiving niclosamide plus PD-L1 Ab treatment showed a more significant delay in tumor growth (Fig. 2a-c) and prolonged survival (Fig. 2d) compared to those receiving monotherapy with niclosamide or PD-L1 Ab. To determine whether niclosamide enhanced anti-PD-L1-mediated promotion of T cell cytotoxic activity, we further examined the tumor infiltrating lymphocytes (TILs) and relative activation in tumor tissues derived from mice. Compared with the monotherapy groups, we observed that combined therapy significantly increased the number of CD3+ TILs. In addition, the highest level of Granzyme B, a T cell activation marker, was observed in the combined therapy group (Fig. 2e-h). Collectively, these results demonstrate that niclosamide combined with anti-PD-L1 treatment enhances the number of TILs with compelling anti-tumor immune activity.

### Niclosamide suppresses p-STAT3 and PD-L1 expression in a dose- and time-dependent manner

**Fig. 3:**
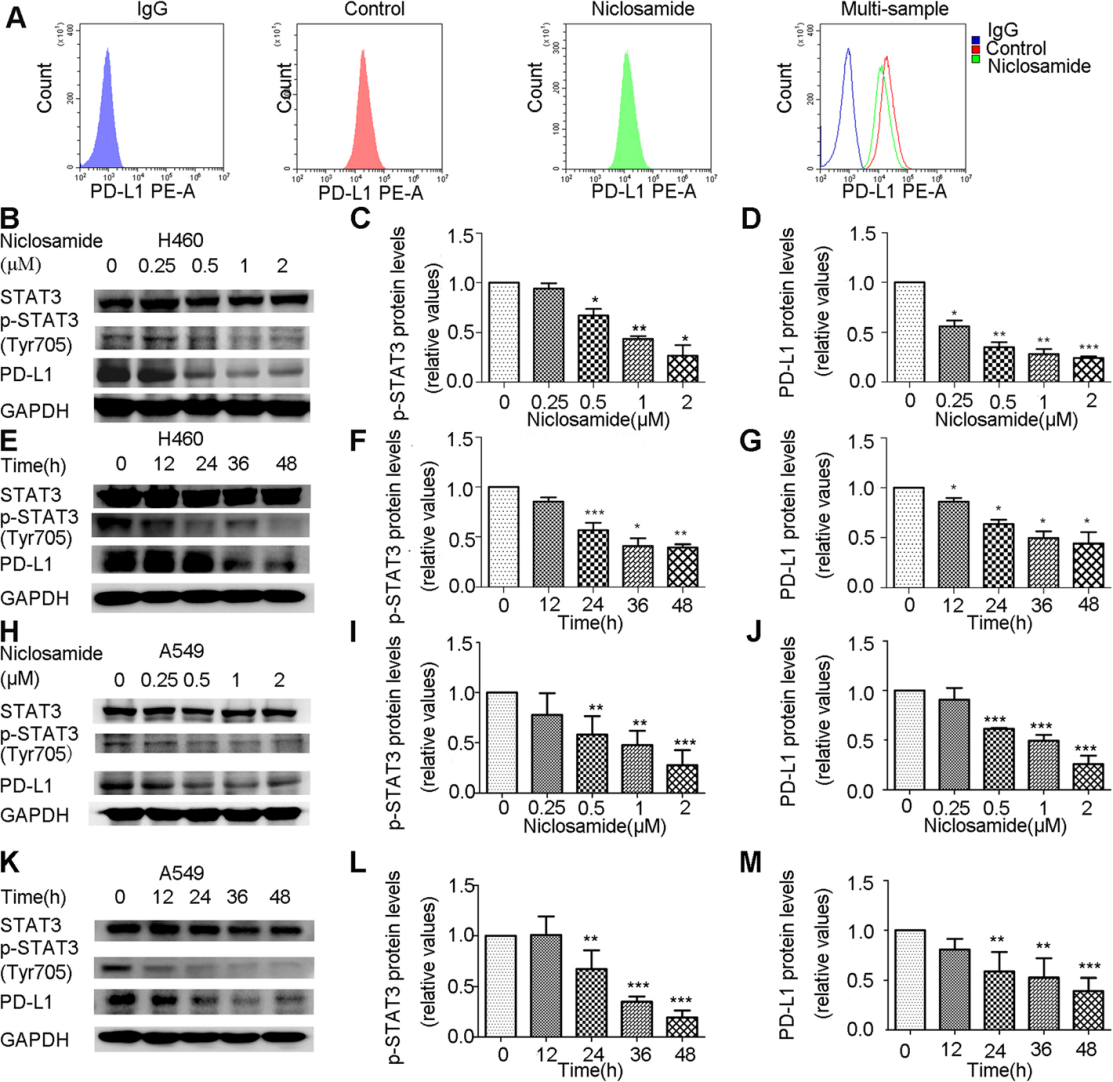
Niclosamide suppress p-STAT3 and PD-L1 expression in a dose- and time-dependent manner. **a** Representative changes in membrane PD-L1 expression, evaluated by flow cytometry analysis on H460 cells treated with the niclosamide. **b**-**d**, **h**-**j** H460 and A549 cells were treated with different concentrations of niclosamide for 48 h, p-STAT3, STAT3 and PD-L1 expression was measured by Western blot. **e-g**, **k**-**m** H460 and A549 cells were treated with 2 μM niclosamide for different time intervals, p-STAT3, STAT3 and PD-L1 expression was measured by Western blot. * *P* < 0.05, ** *P* < 0.01, *** *P* < 0.001. Error bars represent SD of three independent experiments

To further explore the potential mechanism of enhancement of PD-L1 antibody by niclosamide, we evaluated whether niclosamide could have an impact on PD-L1 expression. The maximum niclosamide concentration tested (2 μM) was added to these NSCLC cell lines, which was lower than the IC30. Applying flow cytometry analysis, we observed downregulation of PD-L1 expression after niclosamide treatment for 24 h (Fig. 3a).

We further validated the inhibitory effect of niclosamide on PD-L1 expression. After treatment with differing concentrations of niclosamide, we observed that niclosamide decreased PD-L1 expression as well as STAT3 phosphorylation in a concentration-dependent manner in NSCLC cell lines (Fig. 3b-d, h-i). Moreover, cells treated with 2 μM niclosamide at different time points showed a time-dependent suppression of PD-L1 and p-STAT3 levels (Fig. 3e-g, k-m).

### Niclosamide reduces PD-L1 expression through decreasing STAT3 phosphorylation and its binding to the PD-L1 promoter in nucleus

**Fig. 4:**
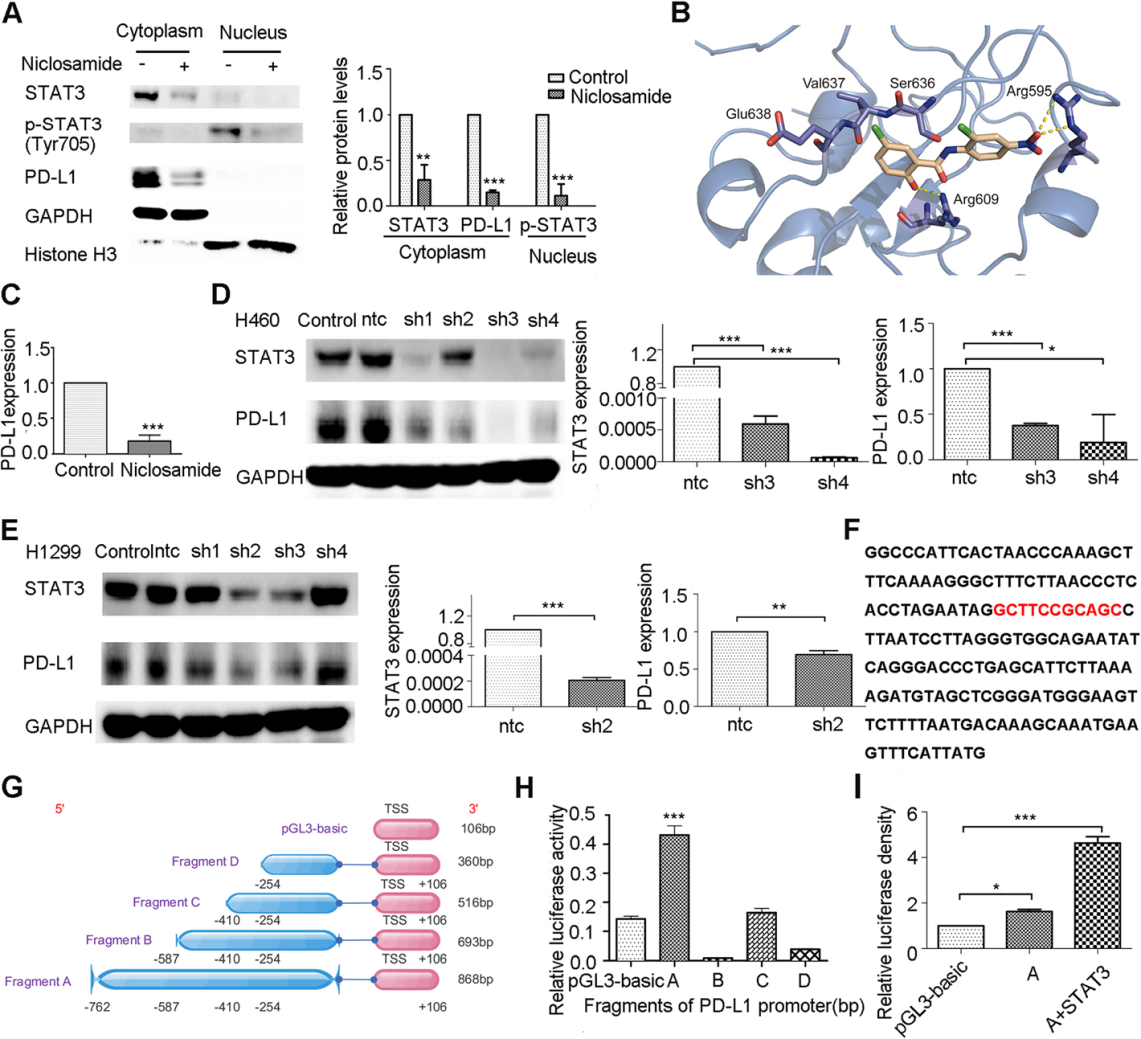
STAT3 increases PD-L1 expression through direct binding to the PD-L1 promoter. **a** The cytoplasm and nuclear translocation of STAT3 analyzed using cell fractionation in H460 cells after niclosamide treatment. **b** The putative binding mode of niclosamide and STAT3. STAT3 was shown as marine Cartoon and key residues was shown as marine sticks. Niclosamide was shown as light orange sticks. Hydrogen bonds were depicted as yellow dashed lines. **c** Relative mRNA expression levels of *PD-L1* were decreased by niclosamide treatment in tumor cells. **d**-**e** Tumor cells expressing shSTAT3 or control were evaluated for STAT3 and PD-L1 expression by qRT-PCR and Western blot. **f** The − 765 to − 587 nucleotide sequence of the 5′-flanking region of PDL1 is shown. Underlined sequences are putative STAT3 transcription factor binding sites, as predicted by PROMO. **g** Overview of the four PD-L1 promoter fragments cloned into pGL3-Basic vector. **h** Luciferase activity measured and normalized according to *Renilla* luciferase activity in 293 T cells transiently transfected individually with the four promoter fragment constructs and empty luciferase vector pGL3-Basic for 48 h. Results are displayed as means ± S.D. of a representative experiment performed in triplicate. **i** Analysis of PD-L1 promoter fragment A construct in 293 T cells transiently transfected with STAT3 for 48 h. Relative luciferase activity was determined as described. Results are represented as means ± S.D. of a representative experiment performed in triplicate. * *P* < 0.05, ** *P* < 0.01, *** *P* < 0.001. Error bars represent SD of three independent experiments

As phosphorylation of STAT3 plays a vital role during its nuclear localization, we isolated nuclear and cytoplasmic fractions from H460 cells treated with niclosamide or not. Data showed, niclosamide reduced cytoplasmic and nuclear STAT3 and its phosphonate expression and decreased cytoplasmic PD-L1 expression (Fig. 4a). To understand the structural basis of the inhibitory effects, the binding mode of niclosamide and STAT3 was investigated. Figure 4b indicates that niclosamide anchors in the phosphotyrosine binding site formed by Lys591, Arg595, Arg609, Ser636, Glu638. Niclosamide is tightly “locked” in the phosphotyrosine binding site via its two ends hydrogen-bonded to Arg595 and Arg609, respectively, which prohibit the binding of STAT3 with its tyrosine phosphorylation receptor, subsequently, inhibits the phosphorylation of Tyr705 (Fig. 4b). Furthermore, the mRNA level of *CD274*, the gene encoding PD-L1, was decreased after niclosamide treatment, indicating that niclosamide induces transcriptional downregulation of PD-L1 (Fig. 4c).

To evaluate whether genetic depletion of STAT3 directly modulates the expression of PD-L1, STAT3 was knocked down using four individual shRNAs (short hairpin RNAs), named sh1–4. Both protein and mRNA expression levels of PD-L1 were potently reduced in STAT3 knockdown tumor cells (Fig. 4d, e).

Given that PD-L1 mRNA levels are clearly decreased by niclosamide, we hypothesized that niclosamide may inhibit the transcription of PD-L1. We predicted STAT3 binding sites present within the PD-L1 promoter using PROMO (http://alggen.lsi.upc.es/) and JASPAR databases (http://jaspar.genereg.net) (Fig. 4f). The sequence of the PD-L1 promoter was cloned into a plasmid vector (pLG3-basic). To establish the major regulatory elements of the PD-L1 promoter, we generated a series of plasmids containing pGL3-basic luciferase elements including either pGL3-basic alone or 4 individual fragments of the PD-L1 promoter (Fig. 4g). We then conducted luciferase assays in 293 T cell lines. The 868 bp PD-L1 promoter fragment showed the highest luciferase activity among all fragments (Fig. 4h), indicating that regulatory sequences present between − 762 and − 587 bp upstream of the PD-L1 transcriptional start site include the primary element(s) responsible for PD-L1 promoter-driven luciferase activity. To explore whether transcription factor STAT3 could bind within this sequence, we utilized PCDNA3.1/STAT3 plasmids and performed co-transfections into 293 T cells. In a reporter assay, we observed that PCDNA3.1/STAT3 notably increased PD-L1 promoter-driven luciferase activity (Fig. 4i). These results suggest that STAT3 directly binds to the PD-L1 promoter to transcriptionally upregulate its expression, and niclosamide attenuates PD-L1 expression through decreasing the p-STAT3 levels of nucleus, and finally reducing its further binding to the promoter of PD-L1.

### Knockdown of STAT3 combined with PD-L1 antibody exhibits enhanced anti-cancer immunity

**Fig. 5:**
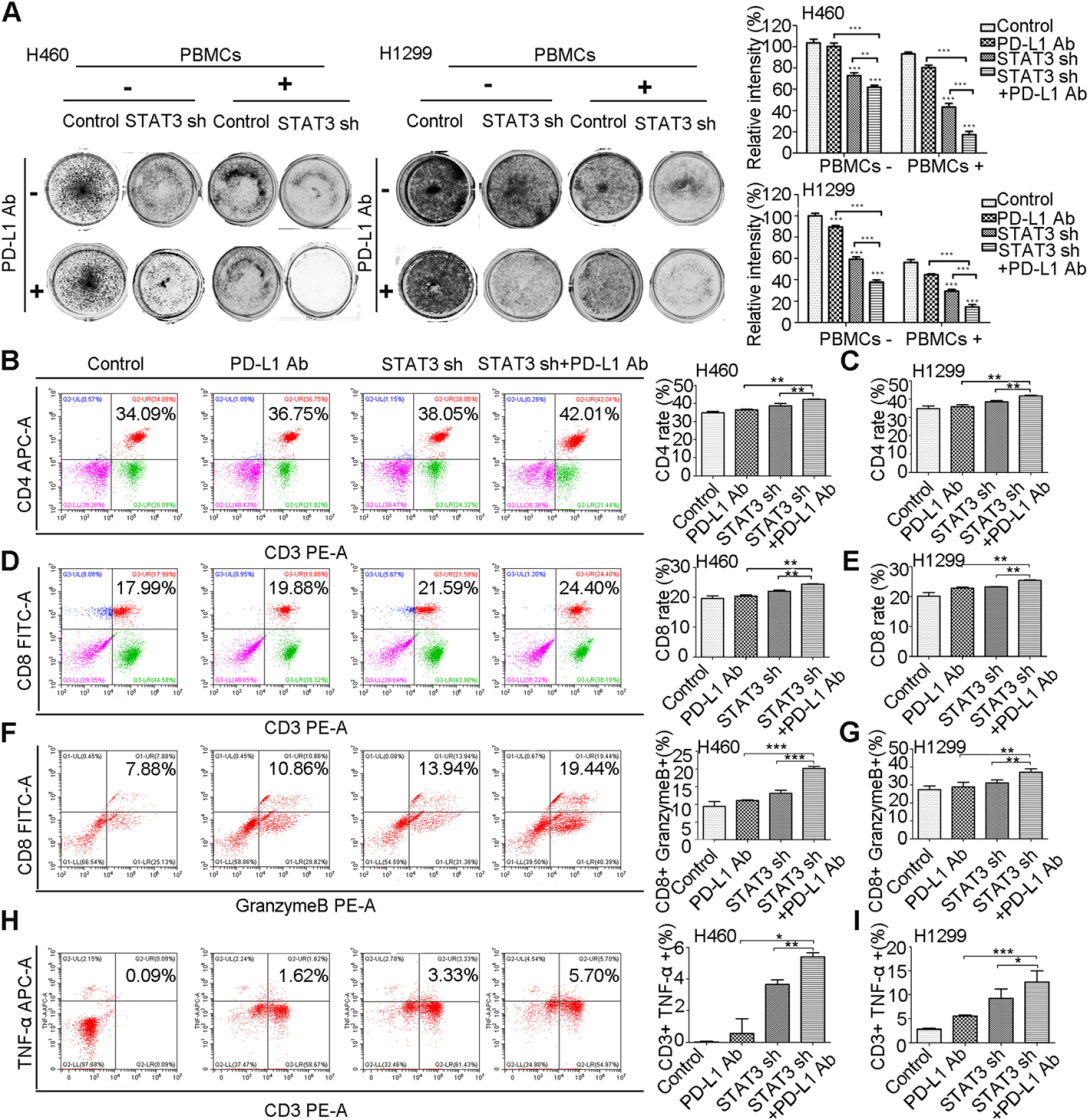
Knockdown of STAT3 combined with PD-L1 antibody exhibits enhanced anti-cancer immunity. **a** T cell cytotoxicity test by colony formation assay. The survival of H460 and H1299 cells with or without genetic downregulation of STAT3, treated with PD-L1 Ab or not, and co-cultured with PBMCs in 24-well plates for 4 days was estimated. Colonies were visualized by Giemsa staining. Survival relative to control is shown. **b**-**e** The effect of STAT3 knockdown H460 and H1299 cells as well as control cells combined with PD-L1 Ab or not on CD4+ and CD8+ populations among CD3+ T cells. Results are presented as means ± S.D. of a representative experiment performed in triplicate. **f**-**g** Intracellular cytokine staining of granzyme B in T cell-mediated tumor cell killing assay in STAT3 knockdown H460 and H1299 cells as well as control cells combined with PD-L1 Ab or not. **h**-**i** Intracellular cytokine staining of TNF-α in T cell-mediated tumor cell killing assay in STAT3 knockdown H460 and H1299 cells as well as control cells combined with PD-L1 Ab or not. Results are presented as means ± S.D. of a representative experiment performed in triplicate. * *P* < 0.05, ** *P* < 0.01, *** *P* < 0.001

Due to the observations that niclosamide downregulates PD-L1 expression dependent on the inhibition of STAT3 phosphorylation, accordingly, we asked whether genetic downregulation of STAT3 in NSCLC cells could influence the response of anti-PD-L1 therapy. After co-culturing with PBMCs, there was a significant decrease in colony formation in STAT3 sh cancer cells with the treatment of PD-L1 Ab than other groups (Fig. 5a), suggesting that genetic downregulation of STAT3 can have positive impact on enhancement of anti-PD-L1 therapy.

Similarly, to demonstrate whether STAT3 sh promoting the efficacy of PD-L1 Ab is associated with an increase in CD4+ and CD8+ T cells as well as the enhanced T cell cytotoxicity, we detected the proportion of CD3 + CD4+ T cells and CD3 + CD8+ T cells using flow cytometry. There was a significant increase in the two kind of T cell populations among PBMCs co-cultured with STAT3 sh tumor cells in combination with anti-PD-L1 (Fig. 5b-e). Importantly, two cytotoxic T lymphocyte function tests were used to measure the generation of TNF-α and the secretion of granzyme B. Levels of TNF-α and granzyme B release were higher in PBMCs after co-culturing with PD-L1-treated STAT3 sh cells than non-treated STAT3 sh and control cells (Fig. 5f-i). Collectively, our results indicate that genetic manipulation of STAT3 promotes anti-tumor immunity by enhancing the proportion of T cells and their capacity to kill tumor cells.

### STAT3 phosphorylation is positively correlated with PD-L1 expression in tumor tissues from patients with NSCLC

**Fig. 6:**
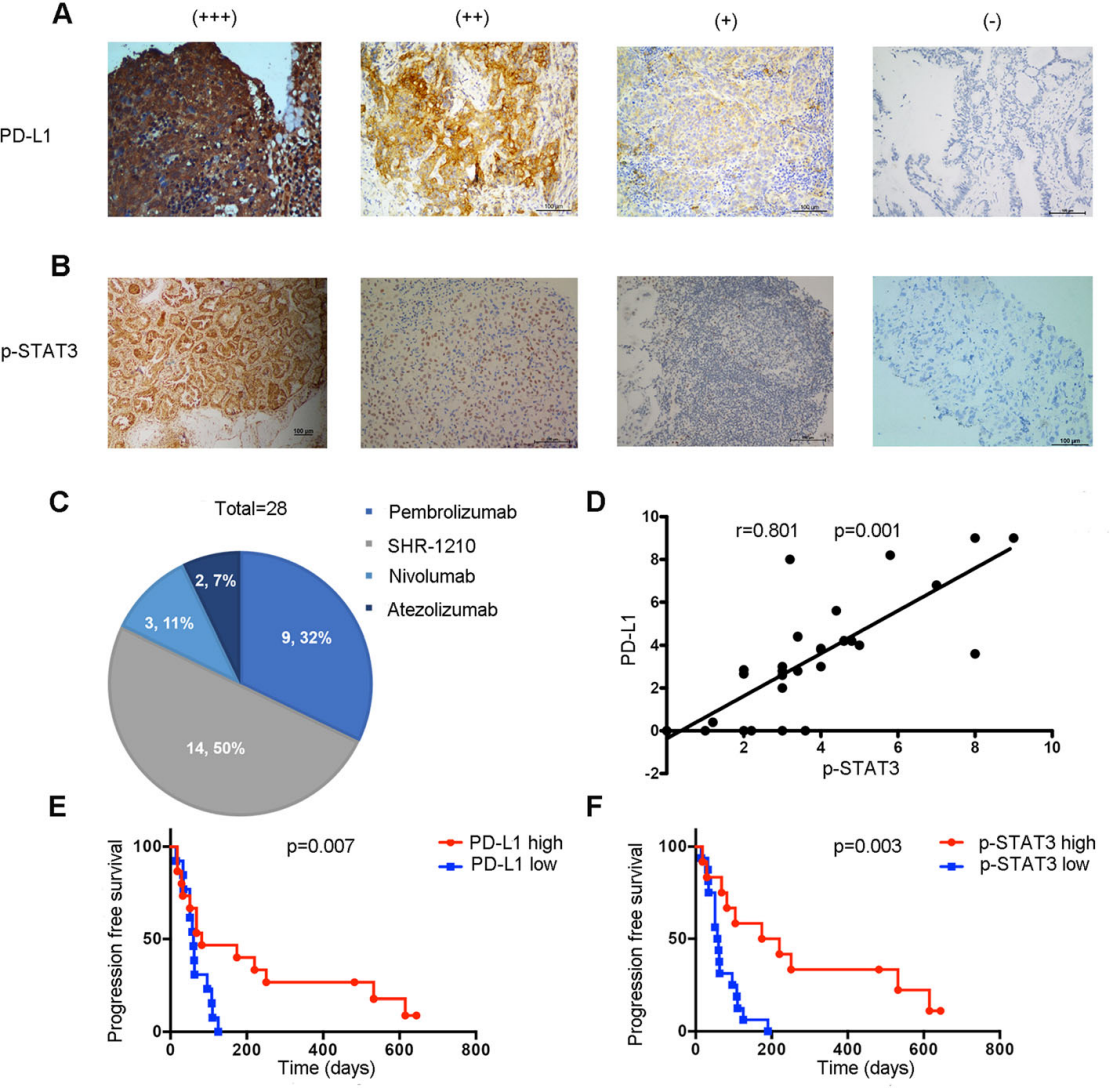
STAT3 phosphorylation positively correlates with PD-L1 expression in tumor tissues from patients with NSCLC. **a**, **b** Representative immunohistochemical staining of PD-L1 and p-STAT3 in human NSCLC. Scale bars = 100 μm. **c** Percent distribution of immunotherapy treatments for included NSCLC patient population. **d** Linear regression analysis of p-STAT3 and PD-L1 immunohistochemical scores in a human NSCLC tissue microarray; *P* < 0.001, r = 0.801. **e**, **f** Kaplan-Meier plots for progression-free survival analysis by optimal cutoff value of PD-L1 and p-STAT3 immunohistochemical scores. Samples were grouped as p-STAT3 high (H-score > 3.8), p-STAT3 low (H-score < 3.8), PD-L1 high (H-score > 2.925), or PD-L1 low (H-score < 2.925)

Given the above findings, we investigated the correlation between the expression of p-STAT3 and PD-L1 in NSCLC, we used immunostaining to detect the levels of these two proteins in 28 NSCLC patients who received immunotherapy (Fig. 6a and b). Of the 28 patients, 50% (14/28) patients received SHR-1210 therapy, 32% (9/28) patients were treated with pembrolizumab, 11% (3/28) patients were administrated with nivolumab, and 7% (2/28) patients received atezolizumab therapy. The percentage distribution of patients receiving various immunotherapeutic agents is shown in Fig. 6c. We found that p-STAT3 had a clear positive correlation with PD-L1 levels (*P* < 0.001, r = 0.801, Spearman rank correlation coefficient; Fig. 6d), suggesting that p-STAT3 exerts a positive effect on PD-L1 expression level in NSCLC.

We then investigated the influence of both p-STAT3 and PD-L1 expression on immunotherapeutic prognosis in patients with NSCLC. First, we calculated the optimal cutoff point according to ROC curves through comparing the sensitivity and specificity of progression-free survival (PFS) prediction. The cutoff expression values were 2.925 and 3.8 for PD-L1 and p-STAT3, respectively. Kaplan-Meier curves and log-rank test were performed. We observed that patients with high PD-L1 level had a longer PFS time than those with low PD-L1 level (median PFS 174 days vs. 60 days; *P* = 0.007) (Fig. 6e). Similarly, patients with high p-STAT3 level had a longer PFS time than those with low p-STAT3 level (median PFS 57 days vs. 174 days; *P* = 0.003) (Fig. 6f). These data show that high expression of PD-L1 and p-STAT3 both predict superior prognosis of immunotherapy.

**Fig. 7:**
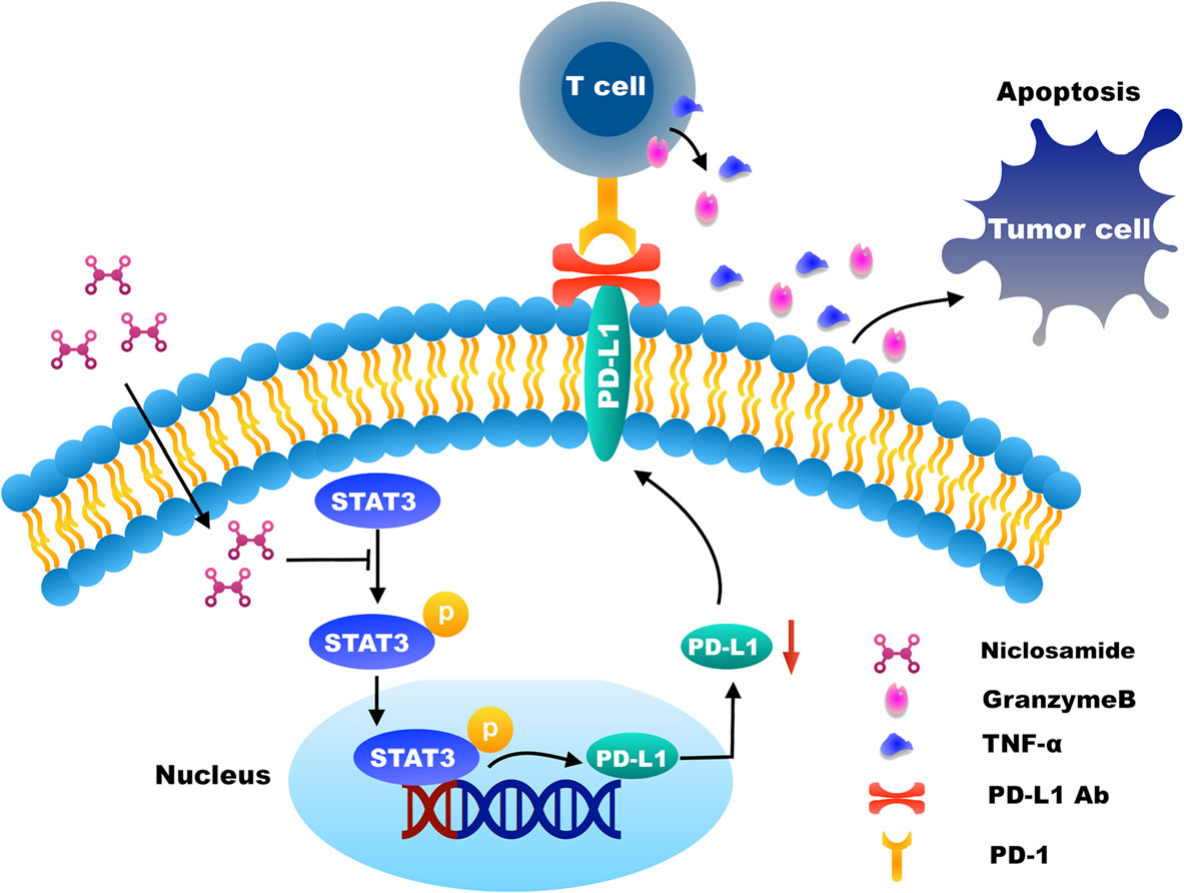
Graphical display of the results

The overview of the mechanism of niclosamide enhanced the antitumor immunity was showed in Fig. 7.

## Discussion

Drugs inhibiting PD-1/PD-L1 signaling have shown promising response in NSCLC treatment. Unfortunately, only approximately 20% of NSCLC patients benefit from immune checkpoint inhibitor as monotherapy. Currently, combinations of various therapies with immunotherapy have been identified as effective and feasible therapeutic approaches to outperform monotherapy. In the present study, we explored whether combination of niclosamide, the antitapeworm drug, and immune checkpoint blockade could elicit augmented anti-tumor response.

We demonstrated that niclosamide could improve anti-tumor immunity to induce increased cancer cell apoptosis when co-cultured with PBMCs. Moreover, our findings establish that niclosamide potentiates anti-PD-L1 blockade response in vitro as well as in syngeneic tumor models, which correlates with an increased content of TILs and enhanced secretion of Granzyme B and TNF-α from cytotoxic T cells. Recent studies have reported that some drugs can enhance T cell infiltration and activation of the tumor microenvironment [29]. These observations suggested that niclosamide in combination with anti-PD-L1 antibody might block tumor growth by downregulating immunosuppressive signaling pathways to achieve maximal anti-tumor immunity.

Considering the strong correlation between PD-L1 expression and T cell exhaustion, we investigated whether niclosamide improved immunotherapeutic effects through regulating the expression of PD-L1. In the current study, we conclude that niclosamide attenuates the PD-L1 expression, which is strongly associated with enhanced cytotoxic T cell activity and anti-tumor immunity. Previous studies have reported that some small molecular drugs similarly promote immunotherapy response through suppression of PD-L1 expression, such as the BET inhibitors [30]. Importantly, we found the antihelmintic drug niclosamide could suppress the expression of PD-L1 through the inhibition of STAT3 phosphorylation and its further binding to the promoter of PD-L1. STAT3 is a key mediator of molecular mechanisms that drive tumor progression and promote immune escape [31]. Furthermore, STAT3 has been identified to be constitutively active and to play a critical role in the development and/or progression of NSCLC [32]. We also observed an augmented anti-cancer immunity when co-culturing the PBMCs and STAT3 sh cancer cells treated with PD-L1 Ab. there was investigation reported that pharmacologic or genetic disruption of STAT3 could enhanced the immunogenicity of cancer cells and further resulted in functional promotion of T cells in B-cell lymphomas [33]. Blockade of STAT3 pathway represents an enticing approach because of its known capability to have an effect on inflammatory status of APC [34], and as shown in current study, to improve the anti-cancer immunity in NSCLC. Therefore, niclosamide, also as a STAT3 inhibitor, may have dual anti-cancer effects on both tumor cells and the tumor-associated immune environment.

Of note, PD-L1 expression in tumors has been regarded as the screening standard in some clinical trials of anti-PD-1/PD-L1 treatment for NSCLC patients [7]. Hence, we next established that p-STAT3 expression is positively associated with PD-L1 protein level in NCSLC specimens, which is consistent with previous studies [17]. Additionally, we demonstrated that PD-L1 expression can serve as a positive predictor for progression-free survival. Similarly, a number of studies have also observed a correlation between PD-L1 expression and the efficacy of PD pathway blockade in patients with various tumor entities [35]. However, The association between PD-L1 expression as evaluated by IHC and clinical response demonstrated inconsistencies across a variety of studies and cancer subtypes [7]. Currently, there is no recommended criterion for PD-L1 IHC testing in guidelines for use of immune checkpoint inhibitor therapy [7]. Unlike the persistent existence of oncogenic driver mutations, the expression of PD-L1 is an inducible and dynamic biomarker which may indicate the relative likelihood of effectiveness, but may not be a predictor of response [35]. Importantly, apart from PD-L1 expression, several other biomarkers have been shown to play a critical role in predicting immune efficacy and guiding patient selection, such as the TMB [36]. The PD-L1 expression of immune effector cells within the tumor microenvironment has also been proposed as a valid predictor of response [37, 38]. Patients with tumor regression despite a lack of PD-L1 expression in both tumor cells and tumor-infiltrating immune cells reveal that PD-L1 expression is merely the tip of the iceberg in predicting clinical response to PD pathway blockade [39]. Overall, we conclude that utilizing PD-L1 expression as a screening criterion for use of treatments targeting the PD-1/PD-L1 axis is not sufficiently established for NSCLC patients.

## Conclusions

In conclusion, these results demonstrated that the combination of niclosamide and PD-1/PD-L1 pathway blockade could improve sensitivity of immunotherapy in vitro and induce significant tumor regression and longer survival in vivo through attenuating PD-L1 expression, increasing tumor infiltrated T cells and activating T cells. Furthermore, the downregulation of PD-L1 by niclosamide was associated with the inhibition of STAT3 phosphorylation. It is envisioned that a combination strategy based on PD pathway blockade coupled with promising therapy will be required to efficiently benefit a large number of patients with specific malignancies. Our results offer an approach of niclosamide in combination with PD-1/PD-L1 Ab, which may deserve additional clinical validation and be further used in future immunotherapeutic strategies to treat patients with NSCLC.

## Supplementary information

**Additional file 1: Table S1.** shRNAs for vectors. **Table S2.** Primers for vector construction. (DOCX 18 kb)

## Data Availability

The data generated and analyzed will be made from the corresponding author on reasonable request. The authenticity of this article has been validated by uploading the key raw data onto the Research Data Deposit public platform (www.researchdata.org.cn), with the approval RDD number as RDDB2019000650.

## Abbreviations

CTLA-4: cytotoxic T lymphocyte antigen 4
LLCcell: Lewis lung cancer cell
NSCLC: non-small cell lung cancer
PBMCs: peripheral blood mononuclear cells
PD-1: programmed cell death protein 1
PD-L1: Programmed cell death 1 ligand
PFS: Progression-free survival
STAT3: signal transducer and activator of transcription 3
TMB: Tumor mutation burden
TNF-α: tumor necrosis factor-α
