# Supplementary material for: Niclosamide, an antihelmintic drug, enhances efficacy of PD-1/PD-L1 immune checkpoint blockade in non-small cell lung cancer
Source: J Immunother Cancer. 2019 Sep 11;7:245. doi: 10.1186/s40425-019-0733-7 (PMC6739982; doi:10.1186/s40425-019-0733-7)
Supplement: Supplementary file 1 — Additional file 1: Table S1. shRNAs for vectors. Table S2. Primers for vector construction. (DOCX 18 kb) [file 40425_2019_733_MOESM1_ESM.docx]

**Additional file 1: Table S1. shRNAs for vectors**

| Gene | shRNA |
| --- | --- |
| NTC | CAACAAGATGAAGAGCACCAA |
| Stat3sh1 | TCTCTGCAGAATTCAA |
| Stat3sh2 | CAGGCTGGTAATTTATATAAT |
| Stat3sh3 | GGCGTCCAGTTCACTACTA |
| Stat3sh4 | AAGTTCATGGCCTTAGGTA |

**Table S2. Primers for vector construction.**

|  | Forward | Reverse |
| --- | --- | --- |
| Stat3/pCDNA3.1 vector | ATGGCCCAATGGAATCAGC | TCACATGGGGGAGGTAGCG |
| p-868 of PDL1 promoter | TGAACCTAACAGCAGGGAAAAC | TTATCATTTTCTAGGCTGGGTG |
| p-693 of PDL1 promoter | AGCCCTGTTTAAGTGTTCTCTG | TTATCATTTTCTAGGCTGGGTG |
| p-516 of PDL1 promoter | CAGAGTGGTGGTACGAAAAGAG | TTATCATTTTCTAGGCTGGGTG |
| p-360 of PDL1 promoter | GCTTCTAAAGGGTACACTGGAG | TTATCATTTTCTAGGCTGGGTG |
